# Exploring the anticancer and antioxidant properties of *Lagerstroemia speciosa* bark extract via phytochemical and molecular docking analysis

**DOI:** 10.1016/j.jgeb.2025.100553

**Published:** 2025-08-14

**Authors:** Shahnaz Parvin Sweety, Tahsin Ahmed Rupok, Mst. Shahnaj Parvin, Jaytirmoy Barmon, Mst. Sarmina Yeasmin, Md. Ekramul Islam

**Affiliations:** a Department of Pharmacy, University of Rajshahi, Rajshahi 6205, Bangladesh; b Department of Pharmacy, Manarat International University, Dhaka 1212, Bangladesh; c Bangladesh Council of Scientific and Industrial Research (BCSIR), Rajshahi Laboratories, Rajshahi 6206, Bangladesh

**Keywords:** *Lagerstroemia speciosa*, Antioxidant, Anticancer, FTIR, GC–MS, *In silico* molecular docking

## Abstract

*Lagerstroemia speciosa* is traditionally used for treating diabetes and inflammation; however, its anticancer potential remains unexplored. This study assesses the antioxidant and anticancer activities of *L. speciosa* bark extract, with a focus on Ehrlich Ascites Carcinoma (EAC) cells in mice. The crude methanol extract (CME) was subdivided into n-hexane (NHF), chloroform (CHF), ethyl acetate (EAF), and aqueous (AQF) fractions. In vitro antioxidant assays identified CHF as the most active fraction, exhibiting high phenolic and flavonoid content. CHF and EAF were further investigated for anticancer activity in an EAC-induced mouse model, where CHF significantly (*P* < 0.05) reduced tumor cell count compared to EAF. Phytochemical characterization using FTIR and GC–MS revealed bioactive compounds, including 9-Methoxybicyclo[6.1.0]nona-2,4,6-triene and 9,12-Octadecadienoic acid methyl ester (E,E). Molecular docking studies demonstrated strong interactions between these compounds and key cancer-related proteins, p53 and Topoisomerase-II, suggesting potential anticancer mechanisms. Overall, this study shows that *L. speciosa* bark extract has therapeutic potential, especially for CHF. Bioactive compounds like 9,12-octadecadienoic acid methyl ester (E,E) and 9-methoxybicyclo [6.1.0]nona-2,4,6-triene contribute to the extract’s antioxidant and anticancer effects in EAC models, possibly by modulating p53 and Topoisomerase-II.

## Highlights

• CHF of *Lagerstroemia speciosa* bark showed potent anticancer and antioxidant effects.
• CHF exhibited strong cytotoxicity against EAC cells, reducing tumor cell count significantly.
• High phenolic and flavonoid content contributed to CHF’s superior antioxidant activity.
• Molecular docking confirmed strong CHF interactions with p53 and Topoisomerase II targets.
• GC–MS analysis identified key bioactive compounds with anticancer and antioxidant properties.

## Graphical abstract

**Figure:**
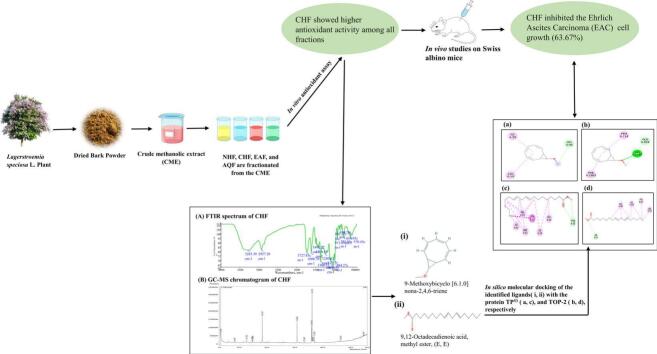

## 1 Introduction

Cancer is defined by the abnormal and unchecked growth of cells in a particular tissue or organ. If left untreated, these cells continue to multiply, forming tumors or other abnormal growths. Malignant changes in a single cluster of cells contribute to cancer development at a localized site before potential metastasis[1], [2], [3]. Cancer continues to be one of the primary global causes of mortality, with 20 million new diagnoses and 9.7 million deaths attributed to the disease in 2022. Statistically, around 20 % of people will experience cancer at some point in their lives, and the mortality rate stands at one in nine for men and one in twelve for women. Out of 183 countries, 177 report cancer among their top three leading causes of death. By 2050, demographic projections estimate 35 million new cases annually^4^.

One of the key characteristics of cancer cells is an imbalance in their redox state, causing them to generate high levels of reactive oxygen species (ROS). While ROS can promote tumor growth, they also exhibit cytotoxic properties^5^. These molecules are generated endogenously by mitochondria, peroxisomes, and inflammatory cells, as well as exogenously by radiation, chemicals, and toxic agents. When the concentration of reactive oxygen species (ROS) exceeds the body’s intrinsic antioxidant capacity, it leads to oxidative damage of DNA, lipids, and proteins. This damage initiates both genetic and epigenetic changes that can disrupt the function of oncogenes and tumor suppressor genes. Moreover, ROS influence the activity of p53, a key tumor suppressor involved in regulating apoptosis. As a result, oxidative stress plays a central role in altering gene regulation, promoting cell growth, and affecting programmed cell death, thereby contributing to cancer development^6^.

Anticancer treatments are designed to specifically target and suppress the growth of cancer cells while minimizing harm to healthy cells. Conventional treatments, including chemotherapy, radiation, and surgery, often cause severe side effects and discomfort^7^. Given ROS's critical involvement in cancer, targeting oxidative stress presents a promising therapeutic approach. Reducing ROS levels can hinder tumor growth and ROS-induced carcinogenesis by promoting oxidative damage and cell death. Cancer patients typically exhibit decreased antioxidant enzyme levels, making antioxidant therapy a potential treatment option. Over time, both enzymatic antioxidants like NADPH oxidase (NOX) blockers and superoxide dismutase (SOD) analogs, along with non-enzymatic antioxidants such as vitamins, N-acetylcysteine (NAC), glutathione (GSH) derivatives, and NRF2 activators, have shown promise in combating cancer[6], [8]. Research suggests that combining antioxidants with chemotherapy and radiation enhances treatment efficacy, reduces tumor size, and improves survival rates^9^.

Medicinal plants are abundant in antioxidants, especially phenolic and flavonoid compounds^10^. These bioactive molecules can inhibit carcinogenesis and inflammatory processes^11^. The anticancer drug paclitaxel, derived from Taxus brevifolia, exemplifies the value of natural products in cancer treatment^12^. At present, more than 60 % of anticancer medications are derived from natural sources, with medicinal plants accounting for around 75 % of them^13^. Naturally occurring antioxidants, particularly plant-derived polyphenols, are gaining recognition for their potential as safe and potent bioactive molecules with ROS-scavenging properties[14], [15].

*Lagerstroemia speciosa* (L.) Pers., also known as Jarul or Queen’s Crape Myrtle, is a medicinal plant belonging to the Lythraceae family and is indigenous to South and Southeast Asia*.* This semi-deciduous tree features obovate leaves, a fluted bole, and slightly flaking bark^16^. Traditional folk medicine practitioners have used *L. speciosa* to treat hypertension, urinary disorders, hyperlipidemia, diarrhea, diabetes, and pain^17^. Its leaves exhibit diuretic, decongestant, anti-diabetic^18^, and anti-obesity properties, while the roots are utilized for treating mouth ulcers, the bark serves as a stimulant and fever-reducing agent, and the seeds possess narcotic properties.[19], [20]. Pharmacological research has revealed that the leaves of *L. speciosa* exhibit a wide range of activities, such as antimicrobial, antioxidant, anticancer, antidiabetic, lipid-lowering, anti-inflammatory, pain-relieving, gastrointestinal, cardiovascular, liver-protective, and kidney-protective effects[21], [17], [22]. Pharmacologically, the bark of *L. speciosa* has received growing attention for its therapeutic properties. Notably, studies have reported that the bark possesses antidiabetic activity, attributed to its ability to enhance insulin sensitivity and reduce blood glucose levels[20], [18]. Furthermore, the bark has shown significant antioxidant activity through the presence of polyphenols and flavonoids, which protect against oxidative damage^21^. Phytochemical studies have reported the presence of several bioactive constituents in the bark, including ellagitannins, flavonoids, and triterpenoids, which contribute to its antioxidant, anti-inflammatory, and potential anticancer properties^23^^,^Wei et al., 2022). Among these, corosolic acid a pentacyclic triterpenoid isolated from the bark has been shown to exert antiproliferative effects by inducing apoptosis, suppressing angiogenesis and inhibiting tumor cell growth in vitro and in vivo cancer models^24^.

Despite these promising properties, the anticancer potential of *L. speciosa* bark has not been extensively explored in Ehrlich Ascites Carcinoma (EAC) models. To address this gap, the present study investigates the antioxidant and anticancer activities of the bark extract, employing a combination of in vitro antioxidant assays, in vivo tumor suppression studies in EAC-bearing mice, and in silico analyses. FTIR and GC–MS were used for phytochemical profiling, while molecular docking targeted cancer-related proteins p53 and Topoisomerase-II to evaluate interaction potential. This integrative experimental and computational approach aims to identify key bioactive compounds and elucidate their mechanisms of action, contributing novel insights into the therapeutic potential of *L. speciosa* bark in cancer research which have not been previously reported for the bark extract in EAC models.

## 2 Materials and methods

### 2.1 Chemicals

DPPH (2,2′-Diphenyl-1-picrylhydrazyl), AAPH (2,2′-Azobis(2-amidinopropane) dihydrochloride), carrageenan, 2-thiobarbituric acid (TBA), and 2-deoxy-D-ribose were sourced from Sigma-Aldrich, Germany. Folin-Ciocalteu reagent, ammonium molybdate, aluminum chloride, and trichloroacetic acid (TCA) were obtained from Merck, Darmstadt, Germany. Catechin and gallic acid were supplied by Wako Pure Chemical Company Ltd., Osaka, Japan. All other chemicals used in the study were of analytical grade unless otherwise specified.

### 2.2 Plant collection and identification

In February 2023, fresh stem bark of *L. speciosa* (L.) Pers. was gathered from various sites around the University of Rajshahi campus in Rajshahi, Bangladesh. The collected plant material was taken to the Phytochemistry Research Laboratory at the University of Rajshahi. A taxonomist confirmed the plant’s identity, and a voucher specimen was deposited in the Department of Botany at the University of Rajshahi for future reference (Accession No.: 29).

### 2.3 Preparation of plant material

Fresh stem bark of *L. speciosa* was collected, thoroughly rinsed with distilled water, and sun-dried for seven days. This was followed by drying in an electric oven at 40 °C for 72 h. The dried material was then ground into a fine powder using a mechanical grinder and stored at room temperature in an airtight container for future use.

### 2.4 Extraction

Approximately 1 kg of dried, powdered stem bark was placed in an amber-colored reagent bottle and immersed in an adequate volume of methanol. The bottle was sealed and left undisturbed for about 15 days, with occasional shaking. After this period, the mixture was first filtered through a clean cotton cloth, followed by filtration using Whatman No. 1 filter paper. The filtrate was then concentrated using a rotary evaporator under reduced pressure at 40 °C to yield a semisolid crude methanolic extract (CME: 40.7 g)^25^.

### 2.5 Fractionation of plant extract

The concentrated crude methanolic extract (CME) of *Lagerstroemia speciosa* bark was separated using a modified Kupchan partitioning method^26^. A total of 40.7 g of CME was fractionated using solvents of increasing polarity, including n-hexane, chloroform, ethyl acetate, and water. This process yielded the following fractions: n-hexane fraction (NHF) – 5.60 g (13.76 %), chloroform fraction (CHF) – 9.30 g (22.85 %), ethyl acetate fraction (EAF) – 8.21 g (20.17 %), and aqueous fraction (AQF) – 17.59 g (43.22 %). All fractions were stored at 4 °C for subsequent in vitro antioxidant and in vivo biological evaluations.

### 2.6 In-vitro antioxidant assay

#### 2.6.1 DPPH free radical scavenging assay

The DPPH (2,2-diphenyl-1-picrylhydrazyl) free radical scavenging activity of the bark extract and its various fractions was assessed using a slightly modified procedure^27^. A range of concentrations (1.25–100 µg/ml) of each sample was prepared in 70 % methanol, and the total volume in each test tube was adjusted to 2 ml with the same solvent. Subsequently, 3 ml of a 0.004 % DPPH solution in 70 % methanol was added to each test tube. As a control, 5 ml of the DPPH solution was used without any sample. Catechin served as the reference standard for evaluating antioxidant potential. Following a 30-minute incubation period in the dark, the absorbance of both test and control samples was recorded at 517 nm using a UV–Visible spectrophotometer (Shimadzu UV Mini-1240, Japan), with 70 % methanol as the blank. The scavenging activity (%) was measured through the following equation-

$$\text{Percentage}\;\left(\%\right)\;\text{of scavenging}\;=\{\left({\text{A}}_{0}-{\;\text{A}}_{\text{1}}\right)/{\;\text{A}}_{0}\}\times \;\text{1}00$$

where A_0_ is the absorbance of the control and A_1_ is the absorbance of the sample.

The IC_50_ value, indicating the concentration needed to neutralize 50 % of DPPH radicals, was determined. All experiments were carried out in triplicate, and the results were averaged.

#### 2.6.2 Hydroxyl free radical scavenging assay

The hydroxyl radical scavenging ability of extract and fractions of *L. speciosa* bark was determined according to the method described by Dnyaneshwar M. Nagmoti (Dnyaneshwar M. Nagmoti, et al, 2012). Various concentrations (500 µL, ranging from 6–100 µg/ml) of the extract and its fractions were prepared in KH_2_PO_4_-KOH buffer (pH 7.4) and placed in separate test tubes. Catechin was used as the reference antioxidant. Following the previously described procedure, the remaining reagents were added in the same order. The absorbance of the test samples was then recorded at 532 nm and compared to a control solution consisting of deoxyribose and buffer. All experiments were conducted in triplicate, and the average values were reported. The scavenging activity (%) was measured through the following equation-

$$\text{Percentage}\;\left(\%\right)\;\text{of scavenging}\;=\{\left({\text{A}}_{0}-{\;\text{A}}_{\text{1}}\right)/{\;\text{A}}_{0}\}\times \;\text{1}00$$

where A_0_ is the absorbance of the control and A_1_ is the absorbance of the sample.

The IC_50_ value denotes the sample concentration required to scavenge 50 % of hydroxyl free radicals. The IC_50_ values were then calculated by plotting percentage inhibitions against concentrations on a linear graph.

#### 2.6.3 Lipid peroxidation inhibition assay

Reactive oxygen species (ROS) induce lipid oxidation and cellular harm. In the lipid peroxidation inhibition assay, the capacity of bark extract and fractions to prevent lipid peroxidation in solution was quantitatively assessed through a previously established method using Bovine brain solution^28^. Different concentrations (100 µL, ranging from 25–500 µg/ml) of extract and its fractions were prepared in Tris-HCl buffer (pH 7.4) and added to individual test tubes, followed by the addition of other reagents as per the described method. The absorbance of the resulting mixtures was measured at 532 nm using a UV–Visible spectrophotometer (Shimadzu UV Mini-1240, Japan). Each experiment was repeated three times, and the results were averaged. Catechin served as the standard antioxidant, while the control contained all reagents except the test sample or standard. The scavenging activity (%) was measured through the following equation-

$$\text{Percentage}\;\left(\%\right)\;\text{of scavenging}\;=\{\left({\text{A}}_{0}-{\;\text{A}}_{\text{1}}\right)/{\;\text{A}}_{0}\}\times \;\text{1}00$$

where A_0_ is the absorbance of the control and A_1_ is the absorbance of the sample.

The IC_50_ value denotes the sample concentration required to inhibit 50 % of lipid peroxidation. The IC_50_ values were then calculated by plotting percentage inhibitions against concentrations on a linear graph.

#### 2.6.4 Reducing power assay

The Fe^3+^ reducing power of the extract and fractions was determined using a previously established method^28^. According to the method, several concentrations (3.125–100 µg/ml) of the extract and fractions of *L. speciosa* bark in methanol were taken in each test tube along with the other reagents. As a reference standard, catechin was used and experiment was conducted in triplicate to ensure reproducibility. The absorbance was taken using a UV–visible spectrophotometer (Shimadzu UV Mini-1240, Japan) at 700 nm against a blank solution containing all the reagents except any test sample. The absorbance is proportion to the reducing power of the test samples.

#### 2.6.5 Total antioxidant assay

The total antioxidant capacity of extract and fractions was determined by the spectrophotometric phosphomolybdenum method^29^. Different concentrations (2 ml, 6–100 µg/ml) of the extract and its fractions dissolved in methanol were mixed with 3 ml of the reagent mixture containing 0.6 M sulfuric acid, 28 mM sodium phosphate, and 1 % (w/v) ammonium molybdate. The mixtures were incubated at 95 °C for 90 min. After cooling, the absorbance of each aqueous solution was measured at 695 nm using a UV–Visible spectrophotometer (Shimadzu UV Mini-1240, Japan), with a blank solution as the reference. The experiment was conducted in triplicate to ensure reproducibility.

### 2.7 *In-vivo* anticancer assay

The Ehrlich Ascites Carcinoma (EAC) model is extensively employed in preclinical cancer research due to its rapid proliferation, high transplantability, and close resemblance to human carcinomas, making it ideal for screening potential anticancer agents (Rajesh et al., 2020). In this study, the anticancer activity of the ethyl acetate (EAF) and chloroform (CHF) fractions derived from the bark extracts was evaluated in male Swiss albino mice. The evaluation followed the method described by Islam et al.^30^, with minor modifications.

#### 2.7.1 Experimental animals

Four-week-old female Swiss albino mice, each weighing between 25 and 30 g, were obtained from the Department of Biochemistry at the University of Rajshahi, Bangladesh. The mice were housed in propylene cages under controlled conditions, maintaining a temperature of 25 ± 2°C, with four animals per cage and a 12-hour light/dark cycle. To promote animal welfare, the cages were furnished with wood chips and nesting materials. The mice had free access to water and a standard diet prepared by ICDDRB. Prior to the experiment, they were acclimatized to the laboratory environment for 14 days. Food and water were withheld for 12 h before the experiment to ensure consistent hydration levels.

#### 2.7.2 Collection of Ehrlich Ascites Carcinoma (EAC) cells

This study utilized transplantable Ehrlich Ascites Carcinoma (EAC) cells. The original inoculum was obtained from the Biochemistry Laboratory at the University of Rajshahi, Bangladesh. In our laboratory, EAC cells were maintained biweekly by intraperitoneal (i.p.) injection of 1 × 10⁶ cells, freshly harvested from donor Swiss albino mice bearing 6- to 7-day-old ascitic tumors. The cells were counted using a hemocytometer and diluted with 0.9 % normal saline to a concentration of 1 × 10⁶ cells per 0.1 ml before injection.

#### 2.7.3 Sample preparation

For in vivo studies, the dried fractions were accurately weighed and dissolved in 0.9 % saline solution to achieve test concentrations of 10 mg/kg and 20 mg/kg body weight for administration in experimental mice. Bleomycin (0.3 mg/kg) was also prepared in saline water to be used as a positive control (reference standard) and only saline water (0.9 % NaCl) was prepared as a negative control.

#### 2.7.4 Experimental procedure and treatment schedule

For this experiment, all the mice were divided into seven groups (I–VII). Group I served as the healthy control group and received only saline solution (0.9 % NaCl) without tumor cell injection. The remaining groups were inoculated intraperitoneally (i.p.) with 1 × 10⁶ actively proliferating EAC cells. Groups II and III acted as the negative and positive controls, receiving daily treatments of 100 µL saline (0.9 % NaCl) and 300 µg/kg Bleomycin, respectively. The test groups IV and V were administered ethyl acetate fraction (EAF), while groups VI and VII received chloroform fraction (CHF) at doses of 10 and 20 mg/kg/day, respectively. Each mouse was given 0.1 ml of the respective treatment solution intraperitoneally every day. On day seven after tumor transplantation, the mice were euthanized, and tumor cells were collected by multiple washes of the peritoneal cavity with 0.9 % saline. The total tumor cell count was determined using a hemocytometer with the trypan blue (0.4 %) exclusion assay to assess viability. The number of viable tumor cells in the treated groups was compared against the negative control group (Group II). The following formula was used to calculate the inhibition of cell growth:

%Cell growth inhibition=(1-Tw/Cw)×100

where, Tw = mean number of tumor cells of Group III, IV, V, VI, and VII, Cw = mean number of tumor cells of Group II

### 2.8 FTIR analysis

FTIR analysis identified the functional groups present in the crude methanolic extract (CME) and chloroform fraction (CHF) and ethyl acetate fraction (EAF). The extracts were prepared using methanol, chloroform, and ethyl acetate as solvents. The FTIR spectra were recorded using a Shimadzu IR Prestige-21 instrument equipped with a diffuse reflectance sampling accessory (DRS-8000). The wavelength range in which the spectra were recorded was 400–4000 cm^−1^^31^. To validate the identification of functional groups, spectra were compared with standard reference spectra from the literature and authenticated compound databases (e.g., Sigma-Aldrich FTIR library). A blank potassium bromide (KBr) pellet was used as the control during FTIR analysis to eliminate background noise and validate peak assignments.

### 2.9 Gas Chromatography-Mass spectroscopy (GC–MS) analysis

The chemical constituents of the crude methanolic extract (CME) were analyzed using GC–MS on an Agilent Technologies system (GC-7890B coupled with MS-5977A MSD) equipped with an HP-5MS column (5 % phenyl methyl siloxane, 30 m × 0.25 mm × 0.25 µm). Helium was used as the carrier gas at a steady flow rate of 1.0 ml/min, and a 1 µl sample was injected. The injector temperature was maintained at 250 °C. The oven temperature was initially held at 40 °C for 2 min, then ramped up to 270 °C at a rate of 5 °C per minute, followed by a 15-minute hold at this final temperature. Mass spectrometry conditions included electron impact ionization at 70 eV and a scan range of 40–700 *m*/*z*. Compounds were identified by comparing their mass spectra with those in the NIST library (Version 2011), using a similarity index (SI) threshold of ≥ 80 % for compound acceptance. Peaks with lower similarity values were excluded to ensure accuracy and confidence in compound identification. A solvent blank was injected before the sample to ensure system cleanliness and to validate that no carryover or contamination affected the interpretation of compound peaks.

### 2.10 Molecular docking analysis

#### 2.10.1 Selection and preparation of the target proteins

p53 (PDB ID: 5ZCJ) and Topoisomerase-II (PDB ID: 1AB4) were selected as molecular targets due to their critical involvement in cancer biology, particularly in Ehrlich Ascites Carcinoma (EAC). p53 acts as a tumor suppressor by regulating DNA repair, apoptosis, and cell cycle arrest, while Topoisomerase-II plays a vital role in DNA replication and chromosomal segregation, making it a well-established target in anticancer drug development (Vousden et al., 2007; Nitiss et al., 2009).

The three-dimensional (3D) crystal structures of the selected target proteins, p53 (PDB ID: 5ZCJ) and Topoisomerase-II (PDB ID: 1AB4), were retrieved from the Protein Data Bank (https://www.rcsb.org/pdb/home/home.do) in PDB format. To prepare the proteins for molecular docking, water molecules and non-standard residues (heteroatoms) were removed, and polar hydrogen atoms were added using Biovia Discovery Studio 2021 (v21.1.0.20298). This step helps minimize non-specific interactions and prevents any interference at the active binding sites during docking simulations. The cleaned protein structures were then saved in PDB format and imported into PyRx software, where they were converted to PDBQT format to facilitate the docking analysis.

#### 2.10.2 Preparation of ligands

Ligand preparation for docking involved the addition of hydrogen atoms and structural optimization. The compounds identified through GC–MS analysis were chosen as ligands, and their three-dimensional (3D) structures were obtained in SDF format from the PubChem database. These files were then converted into PDB format using Open Babel software.

#### 2.10.3 Molecular docking setup and analysis

Molecular docking simulations were performed using AutoDock Vina via the PyRx 0.8 software suite. Ligand structures were retrieved from the PubChem database, then optimized and energy minimized using the Universal Force Field (UFF) before being converted to PDBQT format.

The 3D crystal structures of the target proteins—p53 (PDB ID: 5ZCJ) and Topoisomerase-II (PDB ID: 1AB4)—were prepared using Biovia Discovery Studio 2021. All water molecules and heteroatoms were removed, and polar hydrogen atoms were added. The cleaned protein structures were then imported into PyRx and converted to PDBQT format for docking. A grid box was defined around the active site of each protein by specifying the X, Y, and Z coordinates: p53 (5ZCJ): Grid center (X = 15.6, Y = –20.4, Z = 32.7), dimensions: 25 × 25 × 25 Å. Topoisomerase-II (1AB4): Grid center (X = 45.2, Y = 18.3, Z = –11.6), dimensions: 24 × 24 × 24 Å.

The exhaustiveness parameter was set to 8, and the empirical scoring function of AutoDock Vina was used to predict binding affinities (ΔG, kcal/mol). After docking, the top-ranked conformations were selected based on binding energy, and protein–ligand interactions were visualized using Biovia Discovery Studio to analyze binding residues and bonding types (e.g., hydrogen bonds, hydrophobic interactions).

### 2.11 Statistical analysis

All statistical analyses and graphical representations in this study were performed using IBM SPSS Statistics for Windows, version 27.0.1 (SPSS Inc., Chicago, IL), GraphPad Prism version 8.0.1, and Microsoft Excel 2021. Results were expressed as the mean ± standard deviation (SD). To determine the significance of differences among group means, one-way ANOVA followed by Tukey's Honestly Significant Difference (HSD) post hoc test was applied. A p-value of less than 0.05 was considered statistically significant.

## 3 Results

### 3.1 *In vitro* antioxidant assay

**Fig. 1:**
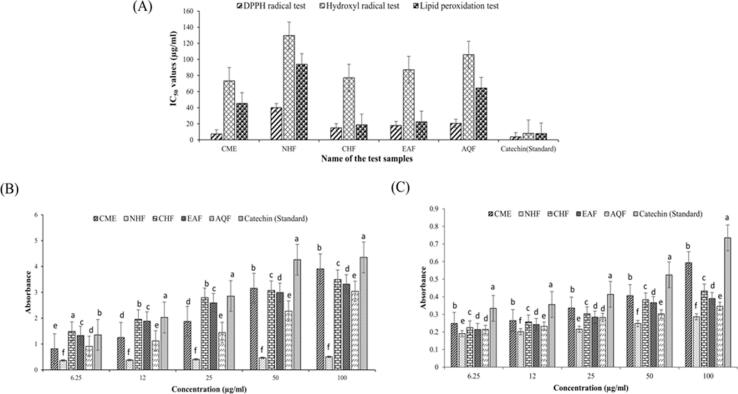
In vitro antioxidant activities of *L. speciosa* bark extract and its solvent fractions. (A) DPPH radical scavenging activity; (B) Hydroxyl radical scavenging activity; (C) Lipid peroxidation inhibition; (D) Ferric reducing power (FRP); (E) Total antioxidant capacity. Results are expressed as IC_50_ values (μg/mL) for DPPH, hydroxyl radical scavenging, and lipid peroxidation assays, and as absorbance values (mean ± SD, n = 3) at 100 μg/mL for FRP and total antioxidant assays. Catechin (CAT) was used as the standard antioxidant. Different letters (a–f) above bars indicate statistically significant differences between groups (P < 0.05, ANOVA followed by Tukey’s post hoc test). Abbreviations: CME – crude methanol extract; NHF – n-hexane fraction; CHF – chloroform fraction; EAF – ethyl acetate fraction; AQF – aqueous fraction.

The antioxidant profile of the extract and fractions were evaluated to assess the anticancer properties for the further study protocol. To estimate the in-vitro antioxidant properties, the DPPH radical scavenging, hydroxyl radical scavenging, lipid peroxidation inhibition, iron-reducing power, and total antioxidant assay were carried out step-by-step. Among all the fractions, CHF showed the highest antioxidant activity among all four fractions followed by EAF, AQF, and NHF in DPPH radical scavenging and hydroxyl radical scavenging assay (Fig. 1A). The IC_50_ value CHF showed 14.94 µg/ml and 77.06 µg/ml in DPPH and hydroxyl radical scavenging assay, respectively (Table 1).

**Table 1:** *In vitro* antioxidant activity of CME and fractions of *L. speciosa* bark and standard.

Name of sample	DPPH radical scavenging assay, IC_50_ (µg/ml)	Hydroxyl radical scavenging assay, IC_50_ (µg/ml)	Lipid peroxidation inhibition assay, IC_50_ (µg/ml)	Ferric-reducing power (FRP) capacity (Absorbance for 100 µg/ml, mean ± SD)	Total antioxidant assay (Absorbance for 100 µg/ml, mean ± SD)
CME	7.33	73.25	45.30	3.905 ± 0.0040	0.593 ± 0.0021
NHF	39.95	129.65	93.82	0.508 ± 0.0036	0.286 ± 0.0060
CHF	14.94	77.06	18.74	3.498 ± 0.0050	0.433 ± 0.0035
EAF	17.78	87.08	22.33	3.318 ± 0.0015	0.390 ± 0.0061
AQF	20.38	105.85	64.38	3.039 ± 0.0012	0.346 ± 0.0042
Standard (Catechin)	3.72	7.96	7.66	4.351 ± 0.0025	0.735 ± 0.0046

All values are expressed as mean ± SD (n = 3), SD = Standard deviation

In the lipid peroxidation inhibition assay, CHF showed the IC_50_ value of 18.74 µg/ml which indicates its highest antioxidant capacity among all four fractions followed by EAF, AQF, and NHF (Table 1, Fig. 1A). In the ferric-reducing power (FRP) and the total antioxidant assay, CHF gave an absorbance of 3.498 ± 0.0050 and 0.433 ± 0.0035 at 100 µg/ml concentration, respectively, and showed the highest antioxidant power among all the fractions followed by EAF, AQF, and NHF (Table 1, Fig. 1B,1C).

### 3.2 *In vivo* anti-cancer assay

**Fig. 2:**
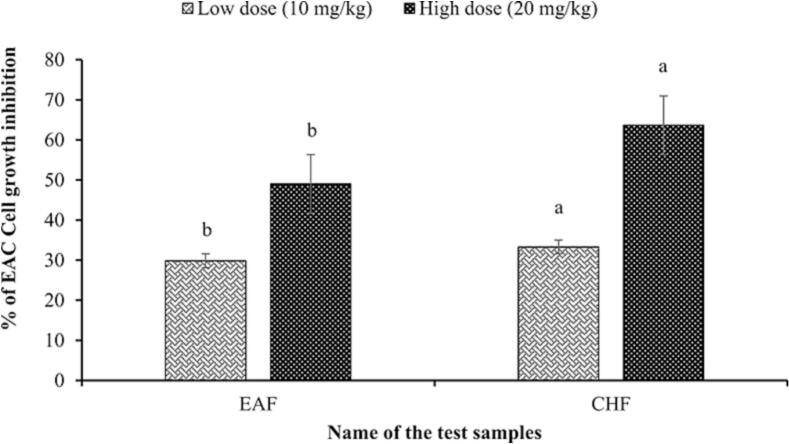
In vivo anticancer activity of both ethyl acetate and chloroform fraction. Inhibition of cell growth, %: EAF:29.84 ± 1.9981 (at 10 mg/kg dose), 49.03 ± 2.8288 (at 20 mg/kg dose); CHF: 33.29 ± 2.5468 (at 10 mg/kg dose), 63.67 ± 0.1863 (at 20 mg/kg dose). Results are expressed as mean ± SD (n = 5). Means with different letters (a,b) differ significantly (P < 0.05).

Ehrlich Ascites Carcinoma (EAC) cell is the tumor cell that transforms itself into a growing cancerous cell in the body of the experimental mice. CHF was selected based on lowest IC_50_ values in DPPH, hydroxyl, and lipid peroxidation assays. Among the tested fractions, the chloroform (CHF) and ethyl acetate (EAF) fractions exhibited the strongest antioxidant activities based on lowest IC_50_ values in DPPH, hydroxyl, and lipid peroxidation assays. Although the crude methanol extract (CME) was also evaluated and showed the highest IC_50_ (indicating lower antioxidant potency), it was not selected for in vivo experiments as it represents the unpartitioned mother extract. Therefore, CHF and EAF were prioritized for further in vivo anticancer evaluation due to their superior antioxidant potential. In the *in-vivo* mice model study of EAC cell growth inhibition assay, the study shows CHF has potential anti-cancer properties followed by EAF compared to the control group. CHF and EAF showed 33.29 ± 2.5468 % and 29.84 ± 1.9981 % of EAC cell growth inhibition at low doses (10 mg/kg). At high doses (20 mg/kg), CHF showed the highest inhibition (63.67 ± 0.1863 %) than EAF (49.03 ± 2.8288 %). Bleomycin, known as a potential anticancer agent was used as a reference standard and showed the maximum EAC cell growth inhibition (88.12 ± 0.6300 %) at the dose of 0.3 mg/kg. The effects of the test compound and bleomycin on EAC cell growth on day six after the tumor transplantation are shown in Table 2 (Fig. 2).

**Table 2:** Effects of the test samples on EAC cell growth inhibition.

Treatment Group	Dose, mg/kg (i.p.)	No. of EAC cells in mouse on day 6 after tumor cell inoculation × 10^7^	Inhibition of cell growth, %
Gr-II: Control (EAC cell-bearing mice)	−-	(2.512 ± 0.0507)	−-
Gr-III: Bleomycin (antibiotic)	0.3	(0.298 ± 0.0128)	88.12 ± 0.6300
Gr-IV: EAF (low dose)	10	(1.763 ± 0.0852)	29.84 ± 1.9981
Gr-V: EAF (high dose)	20	(1.279 ± 0.0450)	49.03 ± 2.8288
Gr-VI: CHF (low dose)	10	(1.675 ± 0.0301)	33.29 ± 2.5468
Gr-VII: CHF (high dose)	20	(0.913 ± 0.0230)	63.67 ± 0.1863

All values are expressed as mean ± SD (n = 5), SD = Standard deviation, EAF = Ethyl acetate fraction, and CHF = Chloroform fraction.

### 3.3 FTIR spectral analysis

**Fig. 3:**
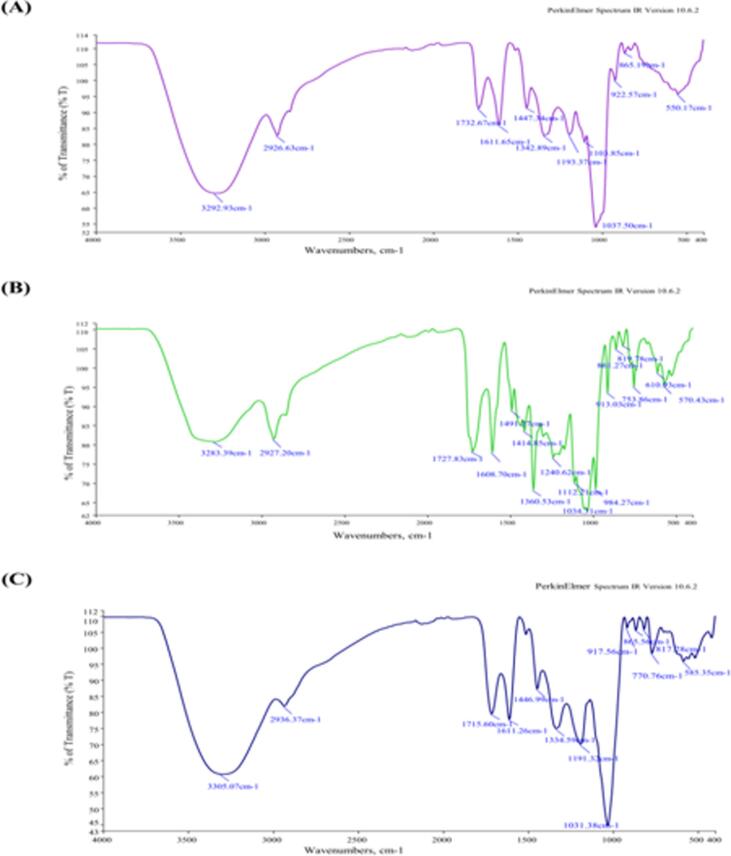
FTIR spectra of *L. speciosa* (A) Crude methanol extract (CME), (B) Chloroform fraction (CHF), and (C) Ethyl acetate fraction (EAF).

The functional groups of different chemical compounds of L. speciosa were evaluated using FTIR spectral analysis[32], [33]. The FTIR spectrum of crude methanol extract (CME) revealed strong peaks corresponding to alkene, secondary alcohol, ester, α,β-unsaturated ketone, aldehyde, and alcohol functional groups. Specifically, CME showed prominent peaks at 1037.50 cm^−1^ (alkene, cyclohexane ring vibrations) and 1611.65 cm^−1^ (α,β-unsaturated ketone, C=C stretching). Medium-intensity peaks were observed for secondary alcohol (1103.85 cm^−1^), ester (1193.37 cm^−1^), and aldehyde (1732.67 cm^−1^), all exhibiting C=O stretching. A strong broad peak at 3292.93 cm^−1^ indicated O–H stretching of alcohols and phenols (Fig. 3A, Table 3).

**Table 3:** FTIR spectral analysis of crude methanol extract (CME) of *L. speciosa* bark.

**Sample**	**Wave numbers, cm-1**	**Appearance**	**Group**	**Compound class**
CME	550.17	Weak, broad	C-I stretch	Alkyl halides
	865.19	Medium	Skeletal C–C vibrations	Alkyne
	922.57	Medium	P-O-C stretch	Aromatic phosphates
	1037.50	Strong	Cyclohexane ring vibrations	Alkene
	1103.85	Medium	C=O stretching	Secondary alcohol
	1193.37	Medium	C=O stretching	Ester
	1342.89	Medium	O–H bending	Phenol
	1447.34	Medium	C–H bend	Alkene
	1611.65	Strong	C=C stretching	α,β-unsaturated ketone
	1732.67	Medium	C=O stretching	Aldehyde
	2926.63	Medium	C–H stretching	Aalkane
	3292.93	Strong, broad	O–H stretching	Alcohol

CHF	570.43	Medium	C-I stretching	Alkyl halides
	610.93	Medium, narrow	C–H bending	Alkyne
	753.86	Strong	C–H bending	1,2-disubstituted (ortho)
	819.78	Medium	C–H bending	1,4-disubstituted (para)
	861.27	Medium	C–H bending	1,3-disubstituted (meta)
	913.03	Strong, narrow	C–H out-of-plane bend	Alkene
	984.27	Strong, narrow	C–H in-plane bend	Aryl
	1034.31	Strong	C-F stretching	Alkyl halides
	1112.21	Medium, narrow	C-N stretching	Amine
	1240.62	Weak	C-O stretching	Alkyl aryl ether
	1360.53	Strong,narrow	S=O stretching	Sulfonamide
	1414.85	Medium	O–H bending	Carboxylic acid
	1491.27	Medium	− N=O bending	Aromatic nitro compounds
	1608.70	Strong, narrow	C=C stretching	Conjugated alkene
	1727.83	Strong	C=O stretching	α,β-unsaturated ester
	2927.20	Weak, broad	O–H stretching	Alcohol
	3283.39	Strong, broad	O–H stretching	Carboxylic acid

EAF	585.35	Weak	C-I stretch	Aliphatic iodo compounds
	770.76	Medium	C–H bending	1,2,3-trisubstituted
	817.28	Medium	C=C bending	Alkene, trisubstituted
	865.56	Medium	C–H bending	1,2-disubstituted
	917.56	Medium	Skeletal C–C vibrations	Alkyne
	1031.38	Strong, sharp	Cyclohexane ring vibrations	Alkene
	1191.32	Medium, sharp	C-O stretching	Ester
	1334.59	Medium, sharp	O–H bending	Phenol
	1446.99	Medium, sharp	C–H asym./sym. Bend	Alkane
	1611.26	Strong, sharp	C=C stretching	α,β-unsaturated ketone
	1715.60	Strong, sharp	C=O stretching	α,β-unsaturated ester
	2936.37	Weak, broad	O–H stretching	Alcohol, intramolecular bonded
	3305.07	Strong, broad	O–H stretching	Alcohol, intermolecular bonded

These spectral signatures confirm the presence of phenolic and flavonoid compounds in CME, which are associated with antioxidant and anticancer activities. The α,β-unsaturated ketones and aldehydes are known contributors to cytotoxic mechanisms, potentially enhancing anticancer efficacy.

The FTIR spectrum of the chloroform fraction (CHF) showed strong, sharp peaks associated with alkenes, aryl groups, sulfonamides, and conjugated alkenes, identified at 913.03, 984.27, 1360.53, and 1608.70 cm^−1^, respectively. Notable peaks were also recorded at 753.86 cm^−1^ (1,2-disubstituted ortho C–H bending) and 1727.83 cm^−1^ (α,β-unsaturated ester C=O stretching), along with a broad O–H stretching peak at 3283.39 cm^−1^ indicative of carboxylic acids (Fig. 3B, Table 3). These functional groups point to the presence of flavonoids, organic acids, sulfonamides, and phenolic esters, all of which are known for their cytotoxic, anti-inflammatory, and free radical scavenging potential.

The ethyl acetate fraction (EAF) displayed strong and sharp peaks for alkene (1031.38 cm^−1^), α,β-unsaturated ketone (1611.26 cm^−1^), and α,β-unsaturated ester (1715.60 cm^−1^). Medium peaks were seen for ester (1191.32 cm^−1^), phenol (1334.59 cm^−1^), and alcohol (1446.99 cm^−1^), while a strong broad O–H stretching peak at 3305.07 cm^−1^ confirmed the presence of intermolecularly bonded alcohols and phenols (Fig. 3C, Table 3).

Taken together, the FTIR analysis suggests the presence of multiple bioactive compound classes, including phenols, flavonoids, esters, alcohols, ketones, aldehydes, carboxylic acids, and alkaloids**,** which may contribute to the observed antioxidant and anticancer activities of *L. speciosa* bark extracts.

### 3.4 GC–MS analysis

**Fig. 4:**
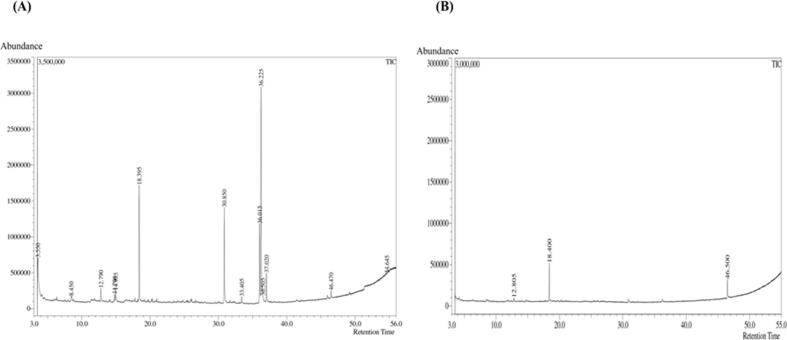
GC–MS chromatogram of the (A) chloroform (CHF) and (B) ethyl acetate fraction (EAF) of *L. speciosa* bark extract.

We performed GC–MS analysis to identify the phytochemicals of chloroform and ethyl acetate fractions of *L. speciosa* bark extracts. The chloroform fraction (CHF) showed 14 peaks in the GC–MS chromatogram and identified 14 phytochemicals, and the ethyl acetate fraction (EAF) showed 3 sharp peaks in the chromatogram and identified 3 phytocompounds with the NIST library 2011 mass spectra library (Fig. 4A,4B). 9,12-Octadecadienoic acid, methyl ester, (E, E) (RT:36.013), 9-Methoxybicyclo [6.1.0] nona-2,4,6-triene (RT:12.782), Hexadecenoic acid, methyl ester (RT:30.850), Phenol, 3,5-bis(1,1-dimethylethyl)- (RT:18.394), 10-Octadecenoic acid, methyl ester (RT:36.227) were the major compounds found in CHF through GC–MS analysis (Table 4). Phenol, 3,5-bis(1,1-dimethylethyl)- (RT:18.396), and 9-Methoxybicyclo [6.1.0] nona-2,4,6-triene (RT:12.791) were identified as major compounds in EAF through GC–MS analysis (Table 5). The phytochemicals found in both CHF and EAF are mainly esters, hydrocarbon (triene), phenols, fatty acid esters, and alcohols.

**Table 4:** Identified Compounds in CHF of *L. speciosa* by GC–MS analysis with reported pharmacological activity.

**SI. No.**	**Retention Time (RT) (min)**	**Area**	**Identified Compounds**	**Molecular Formula**	**Pharmacological Activity**
1	3.547	263,523	3-Methyl-4-(phenylthio)-2-prop-2-enyl-2,5-dihydrothiophene 1,1-dioxide	C_14_H_16_O_2_S_2_	Anticarcinogenic activity^78^
2	8.449	103,328	5,6-Dihydro-5-methyluracil	C_5_H_8_N_2_O_2_	A nucleobase in the nucleic acid of DNA
3	12.782	73,515	9-Methoxybicyclo [6.1.0] nona-2,4,6-triene	C_10_H_12_O	New chemical compound^79^
4	14.790	53,920	1-Tetradecene	C_14_H_28_	Solvent, organic building block
5	14.954	61,367	Benzenemethanol, α-methyl-α-propyl-	C_11_H_16_O	Used in perfumes, flavoring, dye, and laboratory reagent^80^
6	18.394	1,535,761	Phenol, 3,5-bis(1,1-dimethylethyl)-	C_14_H_22_O	Antimicrobial, antioxidant activity^62^
7	30.850	1,402,089	Hexadecanoic acid, methyl ester	C_17_H_34_O_2_	Antioxidant, hypocholesterolemic, nematicide, pesticide, antimicrobial, anti-inflammatory^60^. Anticancer, antihemolytic^81^
8	33.406	39,400	Ethylene brassylate	C_15_H_26_O_4_	Fragrance ingredient^82^
9	36.013	453,654	9,12-Octadecadienoic acid, methyl ester	C_19_H_34_O_2_	Anticancer, antioxidant^83^. Hepatoprotective, antihistaminic, hypocholesterolemic, antieczemic^84^. Analgesic, anti-inflammatory, ulcerogenic properties^85^
10	36.227	1,172,146	10-Octadecenoic acid, methyl ester	C_19_H_36_O_2_	Antibacterial, antifungal, antioxidant, decreases blood cholesterol^61^
11	36.509	2416	2-Butyloxycarbonyloxy-1,1,10-trimethyl-6,9-epidioxydecalin	C_18_H_30_O_5_	Not found
12	37.023	397,315	Methyl stearate	C_19_H_38_O_2_	Antioxidant, cancer preventive, antidiarrheal, anti-inflammatory, pesticide, nematicide, antimicrobial^86^
13	46.467	72,288	Di-n-octyl phthalate	C_24_H_38_O_4_	Antimicrobial, antifouling^87^
14	54.650	51,481	Arsenous acid, tris(trimethylsilyl) ester	C_9_H_27_AsO_3_Si_3_	Used to treat rheumatism, skin diseases, diabetes mellitus, diarrhea, and ulcers^88^

**Table 5:** Identified Compounds in EAF of *L. speciosa* by GC–MS analysis with reported pharmacological activity.

**SI. No.**	**Retention Time (RT) (min)**	**Area**	**Identified Compounds**	**Molecular Formula**	**Pharmacological Activity**
1	12.791	17,741	9-Methoxybicyclo [6.1.0] nona-2,4,6-triene	C_10_H_12_O	New chemical compoundKelany et al.^89^
2	18.396	394,680	Phenol, 3,5-bis(1,1-dimethylethyl)-	C_14_H_22_O	Antimicrobial activity
3	46.497	156,450	Di-n-octyl phthalate	C_24_H_38_O_4_	Antimicrobial, antifouling^90^

### 3.5 Molecular docking analysis

**Fig. 5:**
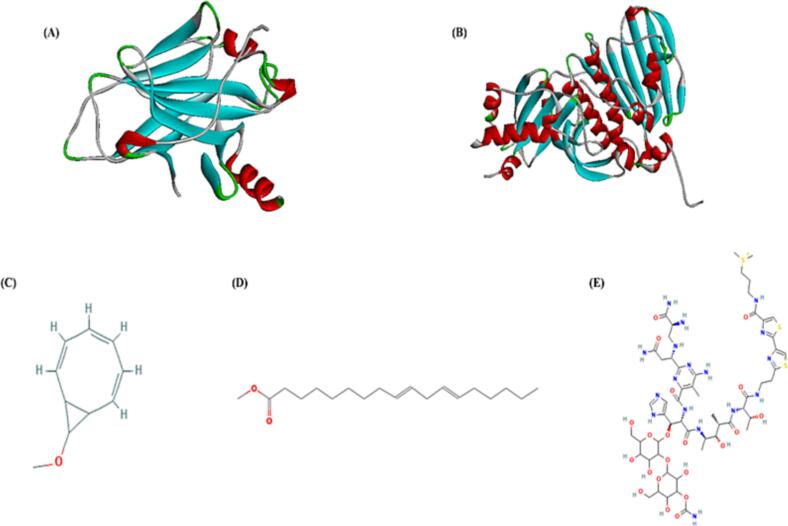
The 3D structure of the proteins (A) p53, (B)Topoisomerase-II, and the 2D chemical structure of the ligands (C) 9-Methoxybicyclo [6.1.0] nona-2,4,6-triene, (D) 9,12-Octadecadienoic acid, methyl ester, (E, E), (E) Bleomycin.

**Fig. 6:**
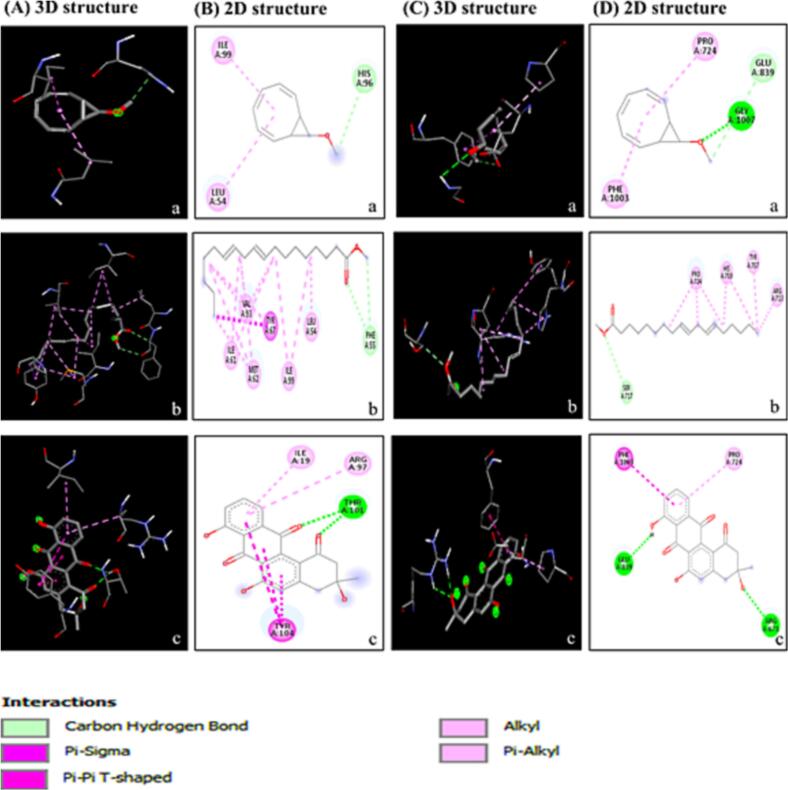
Docking analysis of active site of tumor suppressor protein p53 and topoisomerase-II (TOP-2) with ligands (a) 9-Methoxybicyclo [6.1.0] nona-2,4,6-triene, (b) 9,12-Octadecadienoic acid, methyl ester, (E, E) (c) Bleomycin. (A, C) Three dimensional (3D) and (B, D) Two dimensional (2D) structural interaction between the ligand and protein macromolecules. Multicolor dots and lines indicate different interactions between amino acids of macromolecule and the ligands.

The molecular docking analyses were carried out to evaluate the inclusive molecular interactions of 9-Methoxybicyclo [6.1.0] nona-2,4,6-triene, 9,12-Octadecadienoic acid, methyl ester, (E, E), and Bleomycin with the targeted proteins. Among the identified phytocompounds, 9-Methoxybicyclo[6.1.0]nona-2,4,6-triene and 9,12-Octadecadienoic acid methyl ester were selected for docking based on their relative abundance in GC–MS analysis and reported pharmacological relevance. Their structures are shown in Fig. 5. The ligands exhibited significant interactions with targeted proteins p53, and Toposiomerase-II which was proved by their enumerated binding energies. The more negative the binding free energy means the stronger protein–ligand bond which includes van der Waals, electrostatic, hydrophobic, hydrogen bonds, and other specific interactions^34^. The binding energies of the target proteins p53, and Toposiomerase-II with ligands showed in Table 6. The binding energies of p53 were −5.2, −5.2 and −8.2 kcal/mol for 9-Methoxybicyclo [6.1.0] nona-2,4,6-triene, 9,12-Octadecadienoic acid, methyl ester, (E, E), and Bleomycin and the binding energies of Topoisomerase-II were −6.2, −5.0 and −8.5 kcal/mol for 9-Methoxybicyclo [6.1.0] nona-2,4,6-triene, 9,12-Octadecadienoic acid, methyl ester, (E, E), and Bleomycin respectively. Binding interactions of p53 with ligands revealed hydrogen-bonding interaction with His96 for 9-Methoxybicyclo [6.1.0] nona-2,4,6-triene, with Phe55 for 9,12-Octadecadienoic acid, methyl ester, (E, E), and with Thr101 for Bleomycin. Hydrogen-bonding plays a crucial role which enabling a compound as a potential drug candidate^35^. The p53 also exhibited alkyl-bonding interactions with Leu54, Ile99 for 9-Methoxybicyclo [6.1.0] nona-2,4,6-triene, with Leu54, Ile61, Met62, Ile99, Val93 for 9,12-Octadecadienoic acid, methyl ester, (E, E), and with Ile19, Arg97 for Bleomycin. Binding interactions of Topoisomerase-II with ligands revealed hydrogen-bonding interaction with Gly1007, Glu839 for 9-Methoxybicyclo [6.1.0] nona-2,4,6-triene, with Ser717 for 9,12-Octadecadienoic acid, methyl ester, (E, E), and with Arg673, Glu839 for Bleomycin. The Topoisomerase-II also exhibited alkyl-bonding interactions with Pro724, Phe1003 for 9-Methoxybicyclo [6.1.0] nona-2,4,6-triene, with Arg713, Pro724, Tyr757, His759 for 9,12-Octadecadienoic acid, methyl ester, (E, E), and with Pro724for Bleomycin. The three-dimensional (3D) and two-dimensional (2D) structures are represented in Fig. 6 (A, C) and 6 (B, D), respectively.

**Table 6:** Protein-ligand interactions and binding energies.

**Proteins**	**Ligands**	**Bond type**	**Amino acids involved**	**Binding energy (Kcal/mol)**
**p53**	9-Methoxybicyclo [6.1.0] nona-2,4,6-triene	C–H Bond: 1 Alkyl Bond: 2	H-Bond: His96 Alkyl Bond: Leu54, Ile99	−5.2
	9,12-Octadecadienoic acid, methyl ester (E, E)	C–H Bond: 1 Alkyl Bond: 11 Pi Sigma Bond: 1	H-Bond: Phe55 Alkyl Bond: Leu54, Ile61, Met62, Ile99, Val93 Pi Sigma Bond: Tyr67	−5.2
	Bleomycin	C–H Bond: 2 Pi Alkyl Bond: 2 Pi-Pi Stacked Bond: 3	H-Bond: Thr101 Pi Alkyl Bond: Ile19, Arg97 Pi-Pi Stacked Bond: Tyr104	−8.2

**Topoisomerase-II**	9-Methoxybicyclo [6.1.0] nona-2,4,6-triene	Conventional H-Bond: 1 C–H Bond: 1 Alkyl Bond: 2	H-Bond: Gly1007 C–H Bond: Glu839 Alkyl Bond: Pro724, Phe1003	−6.2
	9,12-Octadecadienoic acid, methyl ester (E, E)	C–H Bond: 1 Alkyl Bond: 7	C–H Bond: Ser717 Alkyl Bond: Arg713, Pro724, Tyr757, His759	−5.0
	Bleomycin	Conventional H-Bond: 2 Pi Alkyl Bond: 1 Pi-Pi T Shaped: 1	H-Bond: Arg673, Glu839 Pi Alkyl Bond: Pro724 Pi-Pi T Shaped: Phe1003	−8.5

## 4 Discussion

Medicinal plants have long been a rich source of therapeutic agents, especially in the development of anticancer drugs. Natural sources have shown significant potential in cancer treatments, offering an alternative to conventional therapies that often suffer from low survival rates, disease recurrence, drug resistance, toxicity, and lack of specificity^36^. Notably, over 50 % of cancer treatment drugs are derived from natural sources^37^. The present study evaluates the antitumor and antioxidant activities of the chloroform fraction (CHF) and ethyl acetate fraction (EAF) of *L. speciosa* bark extracts in Ehrlich ascites carcinoma (EAC) cell-bearing mice.

EAC cells, originally characterized as a rapidly proliferating spontaneous mouse mammary adenocarcinoma, exhibit aggressive behavior in almost all mouse strains^38^. The antitumor effect against EAC cells is typically observed through a decrease in viable cell count and an increase in nonviable cell count, indicating cytotoxicity towards tumor cells^39^. The results of this study demonstrated that CHF, at doses of 10 and 20 mg/kg, exhibited a more pronounced reduction in tumor cell count compared to the tumor control group than EAF. This suggests that CHF has a direct cytotoxic interaction with tumor cells, as anticancer agents administered in the peritoneal cavity are directly absorbed by tumor cells, leading to cell lysis. These findings are consistent with previous studies using similar in vivo models. For example, methanolic extracts of *Annona muricata* leaves significantly reduced tumor volume and ascitic fluid in EAC-challenged mice through pro-apoptotic and antioxidant mechanisms^40^. Similarly, phytochemicals from *Withania somnifera* and *Nigella sativa* have demonstrated notable antitumor effects in EAC-bearing mice, targeting oxidative stress and apoptosis pathways Shah et al.[41], [42]. The tumor inhibition observed with *L. speciosa* extracts is comparable to these plant-based treatments and may be attributed to the presence of triterpenoids, flavonoids, and corosolic acid. These comparisons further validate the therapeutic relevance of *L. speciosa* as a potential anticancer agent. Furthermore, the doses of CHF and EAF administered in mice (10 and 20 mg/kg) correspond to approximately 0.81 and 1.62 mg/kg in humans, respectively, when adjusted using the body surface area normalization method^43^. This suggests a potentially feasible therapeutic window, although further pharmacokinetic and safety studies are warranted before clinical translation

Reactive oxygen species (ROS) are harmful byproducts of oxygen metabolism that can contribute to the development of various diseases, including cancer^44^. ROS homeostasis is maintained by a balance between ROS production and scavenging by antioxidants. Superoxide anions and hydroxyl radicals, common free radicals produced during redox reactions, can lead to oxidative stress and cellular damage unless neutralized by antioxidants[45], [46], [47]. Oxidative stress, characterized by an imbalance between the production of reactive oxygen species (ROS) and antioxidant defenses, plays a critical role in cancer initiation and progression. Excess ROS can damage cellular components, including DNA, proteins, and lipids, thereby promoting mutagenesis, genomic instability, and tumorigenesis. Chronic oxidative stress has been associated with the activation of oncogenic pathways and inhibition of tumor suppressor genes such as p53^48^. Antioxidants, particularly those derived from natural products, counteract these effects by scavenging ROS, restoring redox balance, and modulating key signaling pathways involved in cell proliferation, apoptosis, and inflammation. Numerous plant-derived antioxidants have demonstrated potential in reducing cancer risk by mitigating oxidative DNA damage and enhancing cellular defense mechanisms^49^. In this study, CHF exhibited stronger antioxidant activity than EAF, effectively scavenging free radicals. The DPPH assay demonstrated that antioxidants in *L. speciosa* bark extracts act as hydrogen donors, reducing DPPH radicals[50], [51]. Hydroxyl radicals, known for increasing mitochondrial membrane permeability and inducing apoptosis in tumor cells, are important targets for anticancer agents^52^. CHF effectively scavenged hydroxyl free radicals, reinforcing its potential as an anticancer agent.

Tumor growth is associated with oxidative stress and increased lipid peroxidation, leading to cellular damage in vital organs^53^. Lipid peroxidation can result in oxidative damage to proteins, including the formation of DNA-protein cross-links, which can be measured using lipid peroxidation indices^54^. In this study, CHF inhibited lipid peroxidation in bovine brain lipid samples with an IC_50_ value of 18.74 µg/ml. The ferric reducing antioxidant power (FRAP) assay further confirmed the significant reducing ability of CHF, a key indicator of strong antioxidant potential^55^. Similarly, the total antioxidant assay demonstrated the reducing ability of CHF through the conversion of Mo (VI) to Mo (V), forming a green phosphate/Mo(V) complex at acidic pH (Prieto et al., 1999b). Among the tested fractions, CHF exhibited the most promising antioxidant properties. The strong antioxidant activity of CHF was attributed to its high total phenolic (240.06 ± 0.2556 mg of GAE/g) and flavonoid (190.82 ± 1.1705 mg of CAE/g) content. Phenolic and flavonoid compounds derived from natural sources are known for their antioxidant properties^56^.

To analyze the chemical composition of CHF and EAF, Fourier transform infrared (FTIR) spectroscopy and Gas Chromatography-Mass Spectrometry (GC–MS) analysis were conducted. The FTIR analysis identified various functional groups that correspond to bioactive phytochemicals found in the GC–MS analysis (Althaf Hussain, 2025;^57^^,^Rafe Hatshan^58^. The CME exhibited alkene, secondary alcohol, ester, α, β-unsaturated ketone, aldehyde, and alcohol functional groups, while CHF and EAF revealed additional peaks corresponding to sulfonamide, conjugated alkene, α, β-unsaturated ester, and carboxylic acid groups. The presence of these functional groups strongly correlates with the phytochemicals identified in GC–MS.

The GC–MS analysis of CHF identified 14 bioactive compounds, including 9,12-Octadecadienoic acid, methyl ester, 9-Methoxybicyclo [6.1.0] nona-2,4,6-triene, Hexadecanoic acid, methyl ester, Phenol, 3,5-bis(1,1-dimethyl ethyl)-, and 10-Octadecenoic acid, methyl ester. Similarly, EAF exhibited Phenol, 3,5-bis(1,1-dimethylethyl)- and 9-Methoxybicyclo [6.1.0] nona-2,4,6-triene as its major phytocompounds. The presence of ester groups (C=O stretching at ∼1730 cm^−1^ in FTIR spectra) directly corresponds to the fatty acid methyl esters detected in GC–MS, such as 9,12-Octadecadienoic acid, methyl ester and Hexadecanoic acid, methyl ester. Furthermore, Phenol groups (O–H bending at ∼ 1334 cm^−1^, C-O stretching at ∼1191 cm^−1^) in FTIR align with the presence of Phenol, 3,5-bis(1,1-dimethylethyl)**-** in GC–MS analysis. The alkene and conjugated alkene presence in FTIR (C=C stretching at ∼ 1608–1611 cm^−1^) corresponds to 9-Methoxybicyclo [6.1.0] nona-2,4,6-triene, a hydrocarbon triene identified in GC–MS.

The strong correlation between FTIR and GC–MS results supports the structural characterization of compounds present in the extracts and provides insights into their potential biological activities. Given that many of the identified compounds, such as 9,12-Octadecadienoic acid, methyl ester, possess strong antioxidant and anticancer properties^59^, Hexadecanoic acid, methyl ester is known for its antioxidant, anti-inflammatory, and antimicrobial activities^60^, while Phenol, 3,5-bis(1,1-dimethyl ethyl)- and 10-Octadecenoic acid, methyl ester possess antioxidant and antimicrobial properties[61], [62]. These findings further validate the pharmacological potential of *L. speciosa* bark extracts.

Comparable in vivo anticancer effects have been reported for other plant-based extracts tested against EAC models. For instance, methanolic extracts of Clerodendrum infortunatum and Annona squamosa exhibited significant tumor growth inhibition and improved hematological profiles in EAC-bearing mice^63^. Similarly, ethanolic extracts of Ocimum sanctum and Withania somnifera demonstrated substantial reductions in viable tumor cells and restored oxidative enzyme levels, paralleling the effects observed with CHF in the current study^64^. These comparisons support the view that *L. speciosa* bark extract exhibits anticancer efficacy comparable to other well-established medicinal plants, further underscoring its therapeutic potential.

Given the promising antioxidant and anticancer effects of CHF, further molecular docking studies were conducted to understand its mechanism of action. Molecular docking is a computational technique used to analyze ligand–protein interactions, predict ligand binding mechanisms, and identify the most stable protein–ligand complexes[65], [66]. The tumor suppressor protein P53, which regulates apoptosis, cell cycle arrest, DNA repair, and differentiation, is one of the most critical targets for cancer therapy (Bashir Ahmed^67^. Another key molecular target, Topoisomerase II, is essential for cell viability, DNA replication, and chromosomal segregation. Over expression of Topoisomerase II in tumor cells makes it an attractive target for anticancer drugs that act as catalytic inhibitors^68^.

In this study, the major compounds 9-Methoxybicyclo [6.1.0] nona-2,4,6-triene and 9,12-Octadecadienoic acid, methyl ester (E, E) were used as potential anticancer drug candidates for molecular docking analysis. The standard anticancer drug bleomycin was also included as a reference, as it exerts anticancer effects by inducing DNA damage, inhibiting DNA repair, and promoting apoptosis (Josiah P.^69^. Molecular docking analysis revealed that both test compounds exhibited strong interactions with P53, with a binding energy of −5.2 kcal/mol, compared to Bleomycin's binding energy of −8.2 kcal/mol. Similarly, docking studies with Topoisomerase II showed binding energies of −6.2 kcal/mol and −5.2 kcal/mol for 9-Methoxybicyclo [6.1.0] nona-2,4,6-triene and 9,12-Octadecadienoic acid, methyl ester, respectively, while Bleomycin exhibited a higher binding energy of −8.5 kcal/mol. Lower binding free energy indicates stronger protein–ligand interactions, which are influenced by van der Waals forces, electrostatic interactions, hydrophobic interactions, and hydrogen bonding^34^. The significant hydrogen bonding observed in both test compounds further supports their potential as anticancer agents^35^. Topoisomerase-II plays an essential role in modulating DNA supercoiling during replication and transcription. Compounds that bind to its catalytic domain can interfere with the DNA strand passage or religation step, leading to irreversible DNA double-strand breaks. This action can induce genomic instability and trigger apoptotic cell death in rapidly dividing cells^70^.. The phytocompounds identified in *L. speciosa* bark—particularly those exhibiting docking scores comparable to known inhibitors—may mimic this mechanism by stabilizing the cleavable complex of Topoisomerase-II, thereby promoting cytotoxicity in cancer cells^71^..

Similarly, p53 functions as a central regulator of cell cycle arrest, DNA repair, and apoptosis. Loss or suppression of p53 activity is a common hallmark in many cancers^72^. Natural compounds that bind favorably to the DNA-binding domain of p53 or disrupt its interaction with negative regulators like MDM2 can restore or potentiate its tumor suppressor function^73^. The binding of bark-derived compounds to p53 may enhance its transcriptional activity, upregulate pro-apoptotic genes, and contribute to the observed anticancer effects in EAC models.

While the docking analysis highlighted specific compounds such as 9,12-octadecadienoic acid (Z,Z)-methyl ester for their strong binding affinities with p53 and Topoisomerase-II, it is important to note that the biological effects of *L. speciosa* bark extract are likely the result of synergistic or additive interactions among multiple bioactive constituents. The presence of phenolics, flavonoids, triterpenoids, and fatty acid esters each with known antioxidant and anticancer properties suggests a complex pharmacological profile where compounds may work in concert to enhance therapeutic efficacy[74], [75]. Synergistic effects have been previously reported in plant-based formulations, where mixtures of phytochemicals show higher biological activity than isolated constituents[76], [77]. Therefore, the combined influence of the identified metabolites may significantly contribute to the potent in vivo and in vitro effects observed in this study.

This study is limited by its reliance on a single in vivo cancer model and the absence of mechanistic validation, such as apoptosis assessment or gene expression analysis. Future research should incorporate multiple cancer cell lines and mechanistic investigations to strengthen the findings. Nevertheless, the chloroform fraction (CHF) of *L. speciosa* bark, rich in bioactive compounds with demonstrated antioxidant and anticancer properties, shows considerable promise as a potential anticancer agent. These findings warrant further preclinical and clinical studies to confirm its efficacy and explore its therapeutic potential.

## 5 Conclusion

The chloroform fraction (CHF) of *Lagerstroemia speciosa* bark extract has demonstrated potential antioxidant and anticancer activity as evidenced by its cytotoxicity against tumor cell viability as well as free radical scavenging. These activities are due to its abundance in phenolic and flavonoid compounds, in addition to the existence of bioactive compounds as indicated by GC–MS. Molecular docking was determined to exhibit good binding with significant cancer-related targets p53 and Topoisomerase-II, uncovering possible mechanisms in its cytotoxic activity.

Though such findings show potential for CHF's therapy, further investigations are necessary to establish its activity. These experiments must include the isolation and structural elucidation of pure active constituents, SAR analysis, and mechanistic tests such as caspase induction and apoptosis pathway assays in cultured cancer cell lines. Moreover, safety profiling within cell models and in vivo toxicity would be required to ascertain its suitability for therapeutic use. Development of preclinical formulation for enhancing bioavailability and targeted delivery would further increase its usability in the treatment of cancer.

## Data availability

The synthetic data underlying the present study will be made available upon request.

## Funding source

This research did not receive any specific grant from funding agencies in the public, commercial, or not-for-profit sectors.

## CRediT authorship contribution statement

**Shahnaz Parvin Sweety:** Writing – original draft, Methodology. **Tahsin Ahmed Rupok:** Methodology, Investigation. **Mst. Shahnaj Parvin:** Supervision, Resources, Investigation. **Jaytirmoy Barmon:** Software. **Mst. Sarmina Yeasmin:** Formal analysis. **Md. Ekramul Islam:** Writing – review & editing, Supervision, Resources, Project administration, Conceptualization.

## Ethics approval

The study protocol for the use of animals was approved by the Institutional Animal, Medical Ethics, Biosafety, and Biosecurity Committee (IAMEBBC), Institute of Biological Sciences, University of Rajshahi (Approval no. 09/320-IAMEBBC/IBSc).

## Declaration of competing interest

The authors declare that they have no known competing financial interests or personal relationships that could have appeared to influence the work reported in this paper.
